# Clinicopathological and prognostic significance of Fusobacterium nucleatum infection in colorectal cancer: a meta-analysis

**DOI:** 10.7150/jca.50111

**Published:** 2021-01-15

**Authors:** Shu-chen Huangfu, Wen-bin Zhang, Hao-ran Zhang, Yang Li, Yi-ran Zhang, Jin-lin Nie, Xiao-dong Chu, Chang-shun Chen, Hai-ping Jiang, Jing-hua Pan

**Affiliations:** Department of General Surgery, the First Affiliated Hospital of Jinan University, Guangzhou 510632, China.

**Keywords:** Colorectal cancer, Fusobacterium nucleatum

## Abstract

**Background:** This study aimed to clarify the relationship between F. nucleatum levels and the prognosis of CRC, which is still controversial.

**Methods:** Relevant articles were searched on PubMed, Web of Science, PMC and Embase up to April 7, 2020. Outcomes of interest included clinical characteristics, molecular characteristic and survival analysis. HR (OR), odds ratios (OR) and 95% confidence interval (CI) were calculated to explore the prognostic value and relationship of clinical characteristics of Fusobacterium nucleatum in CRC.

**Results:** A total of 3626 CRC patients from 13 eligible studies were included. High levels of F. nucleatum were associated with worse prognosis, as such parameters as overall survival (OS) (hazard ratio [HR] = 1.40, 95% confidence interval [CI]: 1.40 - 1.63, *P* < 0.0001), disease-free survival (DFS) (HR = 1.71, 95% CI: 1.29-2.26, *P* = 0.0002), and cancer-specific survival (OR= 1.93, 95% CI: 1.42-2.62,* P* <0.0001). F. nucleatum levels were related with T3-T4 stage (OR = 2.20, 95% CI: 1.66-2.91, *P* < 0.00001), M1 stage (OR = 2.11, 95% CI: 1.25-3.56, *P* = 0.005), poor tumor differentiation (OR = 1.83, 95% CI: 1.11-3.03, *P* =0.02), microsatellite instability-high (OR = 2.53, 95% CI: 1.53-4.20, *P* = 0.0003), and KRAS mutation (OR =1.27, 95% CI: 1.00-1.61, *P=*0.05) showed.

**Conclusions:** High levels of F. nucleatum suggest a poor prognosis and are associated with tumor growth, distant metastasis, poor differentiation, MSI-high, and KRAS mutation in CRC patients.

## Introduction

Colorectal cancer (CRC) is the third most frequent malignant tumor with 1.85 million new cases per year around the world based on the statistics in 2019 of the International Agency for Research on Cancer (IARC) of the World Health Organization (WHO) 1. Although great progress has been made in the comprehensive treatment of CRC such as surgery, chemoradiotherapy, anti-angiogenesis, and immunotherapy, the 5-year survival rate is only 10%-20% for CRC patients with distant metastases 2. Therefore, to discover risk factors related to the prognosis is important in improving the survival of CRC patients. Previous studies have reported that epigenetic changes, genetic mutations, chronic inflammation, lifestyle and diet are important factors for the survival of CRC patients 2. Recently, some studies found that intestinal microecological balance is also tight related to the prognosis of CRC 3. A study of Johnson. C et al. showed that colonic mucosal biofilms produced by intestinal flora may affect the development and progression of cancer as a regulator of cellular proliferation and colon cancer growth 4. However, the role of gut microbiota in CRC is still not entirely clear due to its complexity.

Fusobacterium nucleatum (F. nucleatum) is a kind of gram-negative bacilli in the gastrointestinal tract with the ability to express important biofilm tissue behaviors and interactions with host cells through the expression of numerous adhesins 5. Previous studies have proven that it may have a close relationship with occurrence and metastasis of CRC 5-7. A former clinical cohort study demonstrated that high levels of F. nucleatum could be CRC biomarker for prognosis and is associated with a higher CRC-specific mortality 7. On the contrary, some of them indicated that there is no significant correlation between F. nucleatum levels and the prognosis of CRC, especially in the analysis of the clinical survival rate 8. Overall, the correlation between high abundance of F. nucleatum and the clinical and prognosis characteristics of CRC is still controversial.

In view of the above controversial statements, here we investigated this study with an integrated large sample size to clear the prognostic role of F. nucleatum infection in patients with CRC.

## Methods

### Data sources

According to the Preferred Reporting Items for Systematic Reviews and Meta-Analyses (PRISMA Statement) 9, we searched and accessed relevant articles published up to April 7, 2020 through PubMed, Web of Science, Medline, PMC and Embase using MeSH. Key terms included the following: “Fusobacterium nucleatum”, “Fusobacterium spp”, “Fn”, “Colorectal Neoplasm(s)”, “Colorectal Tumor(s)”, “Colorectal Carcinoma(s)”, “Colorectal Cancer(s)”, “prognosis”, “Prognoses” and “Prognostic Factor(s)”.

### Inclusion criteria

In this meta-analysis, the criteria were showed as following for included eligible studies: (1) The articles were original articles, (2) All included studies were controlled clinical studies of CRC patients with complete data, (3) The diagnosis of CRC was based on histology, (4) Subjects of studies were human, and the experimental samples were tumor tissues and surrounding tissues after surgical resection, (5) In the included studies, the DNA content of F. nucleatum in tissues was detected by quantitative polymerase chain reaction (PCR) or 16S ribosomal RNA (16S rRNA) sequencing or other detection methods, and cases were divided into low level and high level for the study according to the median cut-off point amount(2^-ΔCt^) of detectable fusobacteria DNA. The review articles, meeting minutes, letters, and only abstracts were excluded in this meta-analysis to ensure that original data was obtained. For studies with the same research team or with overlapping subjects, we selected the articles with the most comprehensive data.

### Data Extraction

Data for each study were extracted by two independent reviewers, H.F and J. P, and verified using predefined standards. When there were disagreements between two reviewers, it was decided by the third reviewer. The data of the included articles collected were as follows: the first author's name, the year of publication, patient ethnicity, date of birth, sample size for different types (F. nucleatum-high/ F. nucleatum-low), sample type, diagnostic techniques of F. nucleatum, the tumor-node-metastasis (TNM) stage, tumor-associated genes type ( KRAS mutation and BRAF mutation) and microsatellite instability (MSI), survival analysis, and follow-up time. The Engauge Digitizer 4.1 (http://digitizer.sourceforge.net/) was used to extract survival data from Kaplan-Meier curves if the detailed odds ratios (OR) and 95% confidence intervals (CIs) for survival were not directly stated in studies.^ 10^ Multivariate analysis were used if both a univariate and multivariate analysis stated in the same comparison. Any discrepancies were discussed and resolved by consensus. The Newcastle-Ottawa scale (NOS) was used to grade articles and ensure the quality of the included studies 10. The included domains were as follows: the adequacy of case definition, representativeness of the cases, number of cases, ascertainment of exposure, detection method and cutoff, assessment of outcome, and adequate follow-up. A higher score indicates a better methodological quality.

### Statistical Analysis

The meta-analysis was performed by means of Review Manager 5.3 (Cochrane Collaboration, Oxford, UK). As for dichotomous variables, the ORs were calculated, reporting 95% CI. Survival outcomes were summarized by using the generic inverse variance method. Overall survival (OS) was defined as the time from diagnosis until death. Disease-free survival (DFS) was defined as the interval between the initial primary diagnosis of CRC and the first relapse or death. Hazard ratio (HR) and 95% CI were calculated to assess the association between high level of F. nucleatum and survival. A fixed model was performed in aggregating and analyzing for results when *I^2^*< 50%. If *I^2^ >*50%, the random-effects analysis was utilized. The pooled effects were determined by conducting a *Z* test, and the statistical significance was defined as the two-sided *P* < 0.05.

## Results

### Flow Diagram of the Studies Retrieved for the Review

**Figure 1:**
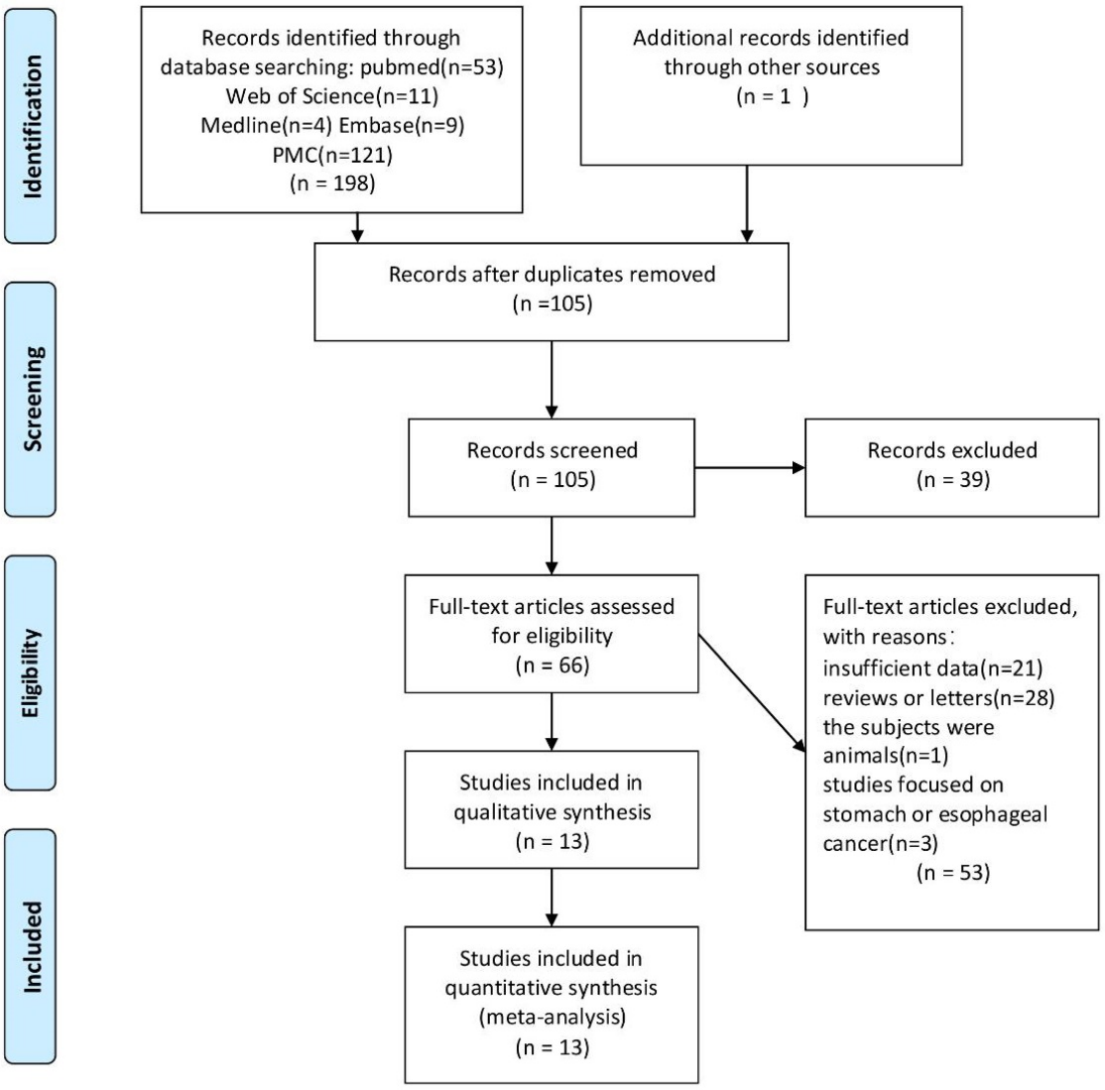
Flow diagram of study selection process.

All 198 articles were identified through PubMed, Web of Science, Medline, PMC and Embase searching after filtration. Among them, 94 articles were duplicated, 39 articles were excluded because of unmatched titles and abstracts, 53 articles were excluded from reading full texts (because of inappropriate objects and incomplete data). The resultant 13 articles 7-8, 11-21 involved 3,690 CRC patients and were included into the meta-analysis. Figure 1 reveals the flowchart of study selection.

### Baseline Characteristics of Included Studies

Table 1 summarizes the main characteristics of the included studies. The included cases originated from 8 countries among North America, Asia, and Europe. All the specimens were tumor tissues after surgical resection. The most commonly used test method for F. nucleatum was the quantitative polymerase chain reaction (qPCR) 7, 12-15, 18-21, and the 16S rRNA method was used in three studies 8, 11, 16 and the droplet digital PCR in one study 17. All cases described in the retrieved articles were divided into two groupsbased on expression level of F. nucleatum DNA. There were 1, 796 in the high-level group and 1, 894 in the low-level group.

### Association between F. nucleatum levels and prognosis of CRC patients

**Figure 2:**
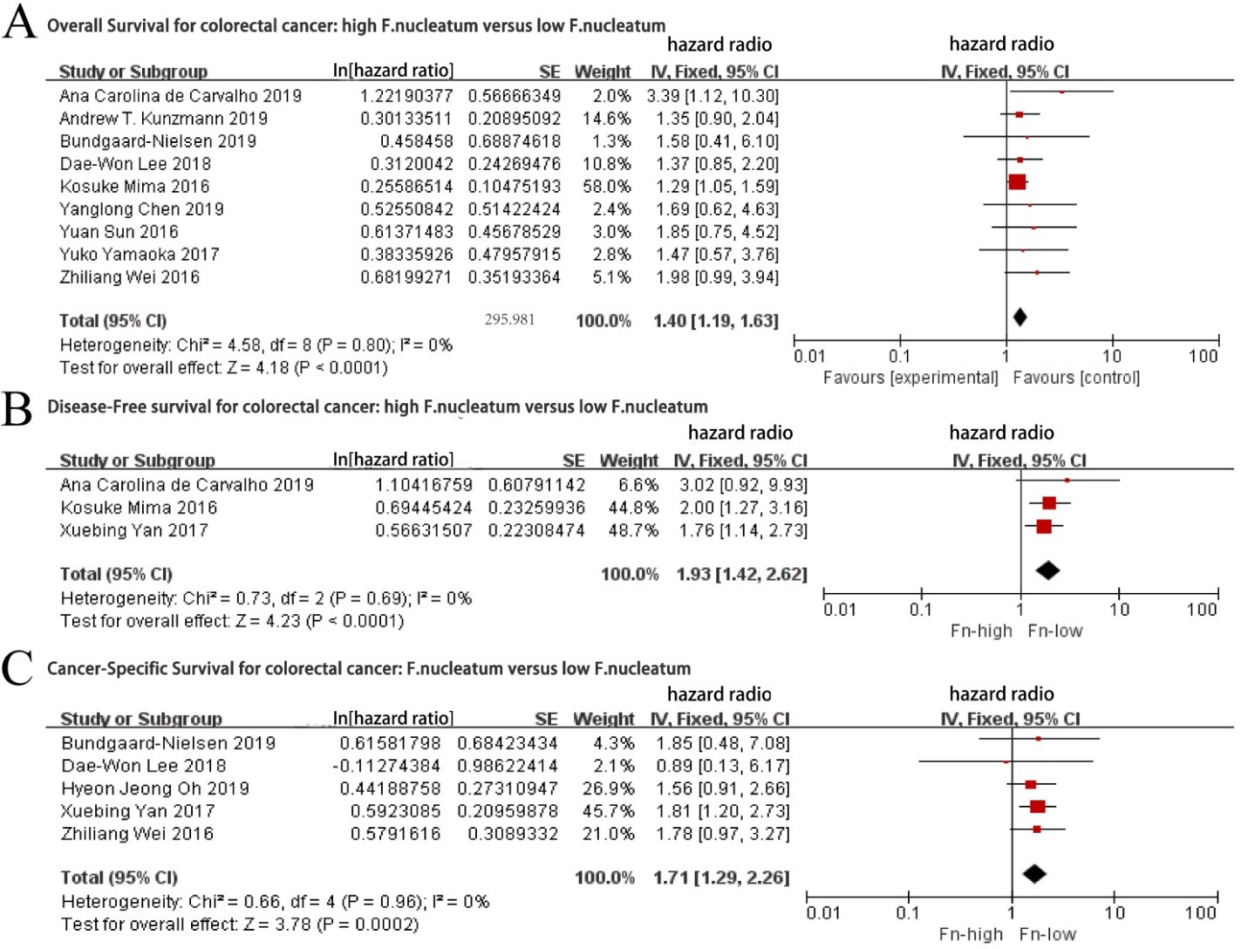
Forest plot for: a. Overall Survival outcomes for colorectal cancer with high level of F.nuleatum versus low level of F.nuleatum; b. Disease-Free Survival outcomes for colorectal cancer with high level of F.nuleatum versus low level of F.nuleatum; c. Cancer-Specific Survival outcomes for colorectal cancer with high level of F.nuleatum versus low level of F.nuleatum.

As the role of F. nucleatum in the prognosis of CRC patients is still controversial, we first analyzed the relationship between F. nucleatum levels and the prognosis of patients with CRC. Nine studies 7-8, 12, 15-17, 19-21 reporting on a total of 2158 patients were pooled for analysis of the association between the F. nucleatum levels and OS. The fixed-effects model was performed as a consequence of low heterogeneity. As shown in Figure 2A, worse OS were revealed in the CRC patients with high F. nucleatum levels (HR= 1.40, 95% CI: 1.40-1.63, *P* < 0.0001), without significant inter-study heterogeneity (*I^2^* = 0%, *P* = 0.80).

Data from five articles 8, 14, 16, 18, 20 with a total of 1270 patients were pooled for analysis of the relationship between the levels of F. nucleatum and DFS. The fixed-effects model was applied as a consequence of low heterogeneity (*I^2^*= 0%, *P* = 0.96). Our results demonstrated that the CRC patients with high levels of F. nucleatum had a worse DFS than those with low levels of F. nucleatum (HR = 1.71, 95% CI: 1.29-2.26, *P* = 0.0002, Figure 2B).

A total of 1498 patients in three studies 7, 18, 21 were included to examine the association between the levels of F. nucleatum and cancer-specific survival (CSS). In this analysis, there was a significant association between high levels of F. nucleatum and poor CSS (HR = 1.93, 95% CI: 1.42-2.62* P* <0.0001), with a low heterogeneity (*I^2^*= 0%, *P* = 0.69, Figure 2C) through the application of the fixed-effects model.

### Association between high levels of F. nucleatum and CRC Clinical Characteristics

As listed in Table 2, data from eleven articles 7, 11-19, 21 included 3413 patients were pooled for analysis of the correlation between the levels of F. nucleatum and primary tumor site in a random-effects model (*I^2^* = 60%, *P*=0.005). The results suggested that there was no correlation between F. nucleatum infection and tumor site (OR = 1.26, 95% CI: 0.91-1.75, *P =* 0.17, supplemental Figure 1A).

A total of 3758 patients in nine studies 7, 11-17, 21 were pooled to examine the association between the levels of F. nucleatum and TNM stage (supplemental Figure 1B). High abundance of F. nucleatum were not associated with the overall TNM stage of CRC (OR= 1.20, 95% CI: 0.96-1.51, *P =* 0.11), with low heterogeneity (*I^2^* = 25%, p = 0.22). However, high levels of F. nucleatum were correlated with high T stages (T3-T4) (OR = 2.20, 95% CI: 1.66-2.91, *P* < 0.00001) and M (M1) (OR = 2.11, 95% CI: 1.25-3.56, *P* = 0.005) stage, without heterogeneity (*I^2^* = 0%). Eight studies reporting on a total of 1445 patients revealed that high F. nucleatum levels were not correlated with N stage (OR = 1.27, 95% CI: 0.98-1.64, *P* = 0.07), with low heterogeneity (*I^2^* = 37%) (supplemental Figure 1C-E). Therefore, our results revealed that there was a relationship between high level of F. nucleatum and large tumor size and distant metastases in CRC.

Furthermore, eight studies 7, 11, 15-19, 21 with a total of 2118 patients reported the association between the levels of F. nucleatum and tumor differentiation (supplemental Figure 1F). As shown in Table 2, high levels of F. nucleatum were significantly associated with poor tumor differentiation (OR = 1.83, 95% CI: 1.11-3.03,* P* = 0.02) in CRC patients, with high heterogeneity (*I^2^* = 60%).

### Association Between high levels of F. nucleatum and molecular characteristics in CRC

In order to further reveal the association between F. nucleatum infection and CRC progression, we analyzed the association between F. nucleatum levels and tumor-specific molecular characteristics. As shown in Table 3, data from six studies 7, 11-14, 21 with 2520 patients demonstrated that high levels of F. nucleatum were significantly associated with MSI-high type CRC (OR = 2.53, 95% CI: 1.53-4.20, *P* = 0.0003), although with a high heterogeneity (I^2^ = 83%, *P* < 0.0001) (supplemental Figure 2A). The correlation between high F. nucleatum levels and KRAS mutation was also found in the fixed-effects model with low heterogeneity (I^2^ = 28%, *P* = 0.23) in six studies 7,11-14, 17 with a total of 2404 patients. The OR was 1.27 with a 95% CI of 1.00-1.61 (*P =* 0.05) (supplemental Figure 2B).

Six studies 7, 11-14, 21 with a total of 2499 patients were pooled for analysis of the association between the levels of F. nucleatum and BRAF mutation. Our results demonstrated that high levels of F. nucleatum were not associated with BRAF mutation in CRC patients (OR = 1.93, 95% CI: 0.91-4.11, *P* = 0.09) (supplemental Figure 2C). In addition, our pool results with three studies^7, 17, 21^ with 1085 patients found that high F. nucleatum levels in CRC tissue had no correlation with MLH1 hypermethylation (OR= 0.78, 95% CI: 0.06-9.93, P = 0.84) (supplemental Figure 2D). A total of 1603 patients in three studies 7, 12, 13 were pooled for analysis of the association between high F. nucleatum levels and PIK3CA mutation through the fixed-effects model, and there was no correlation between and PIK3CA mutation in CRC (OR = 1.21, 95% CI: 0.74-1.97, P = 0.45) (supplemental Figure 2E).

### Sensitivity Analysis

To assess the impact of a single study on the overall meta-analysis, included studies detecting F. nucleatum by quantitative reverse transcription polymerase chain reaction (qPCR) were selected to perform sensitivity analysis (Table 4). The results of the sensitivity analysis are summarized in Table 4. The similar results of those all studies together were revealed in this analyze by using qPCR, including the relationship between F. nucleatum and tumor side, TNM Stage, T stage, N stage, KRAS mutation, OS and DFS in CRC.

### Risk of Bias

**Figure 3:**
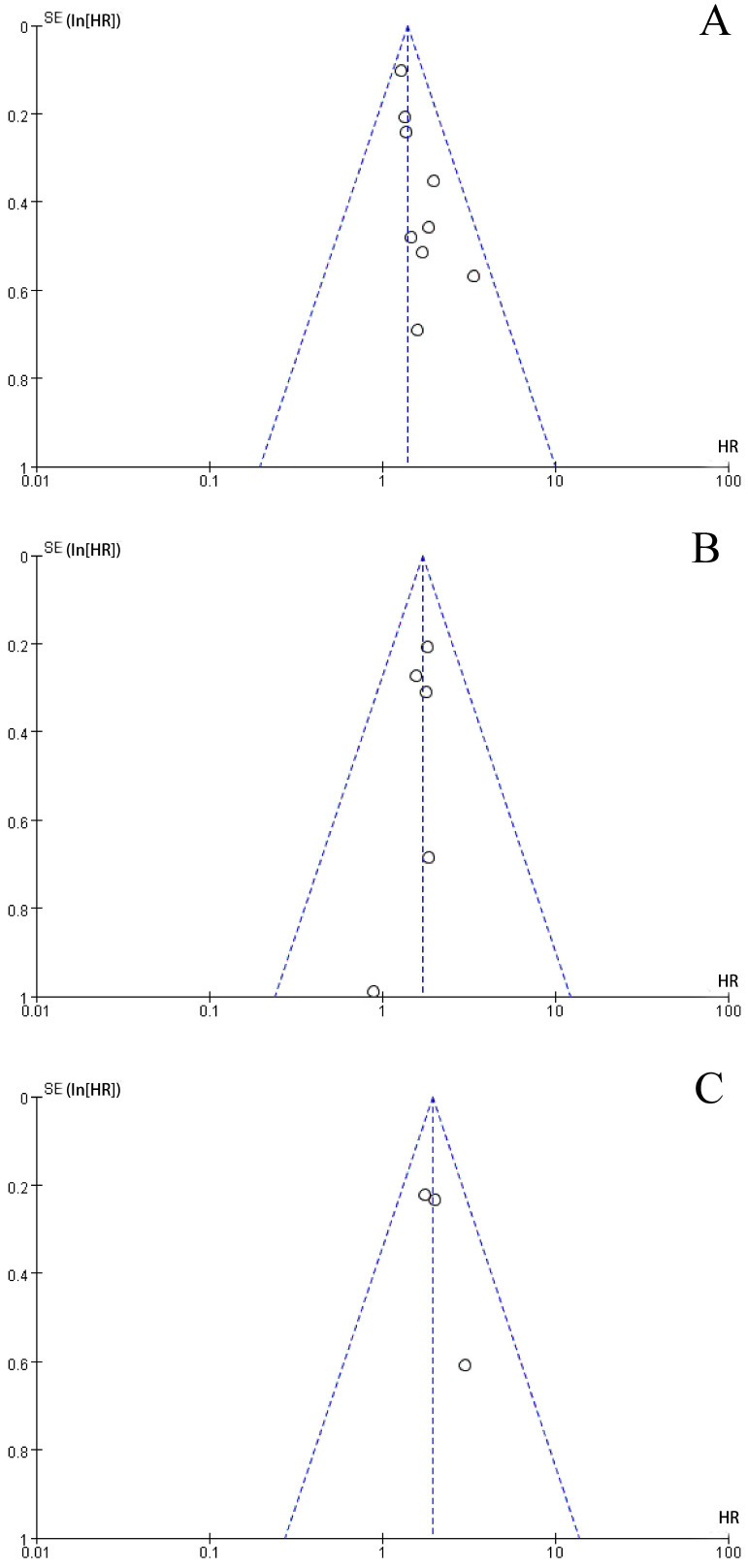
Funnel plots for: a. Overall Survival outcomes for colorectal cancer with high level of F.nuleatum versus low level of F.nuleatum; b. Disease-Free Survival outcomes for colorectal cancer with high level of F.nuleatum versus low level of F.nuleatum; c. Cancer-Specific Survival outcomes for colorectal cancer with high level of F.nuleatum versus low level of F.nuleatum.

The funnel plot was performed to assess publication bias. As shown in Figure 3, the shape of the funnel plots of the main results were roughly symmetrical, without obvious evidence of asymmetry. The funnel plots for main outcomes including OS, DFS, and CSS demonstrated no evidence of publication bias in our study.

## Discussion

This meta-analysis demonstrated that high levels of F. nucleatum are closely related to poor prognosis of CRC patients including OS, DFS, and CSS. Additionally, the correlation between the clinicopathological features of CRC such as tumor site, clinical stage, and tumor differentiation were also observed. We also analyzed the correlation between high levels of F. nucleatum and molecular characteristics of CRC such as MSI and KRAS, BRAF, and PIK2CA mutations, as well as MLH1 hypermethylation. Our results confirmed that high levels of F. nucleatum were significantly associated with MSI-high type and KRAS mutation of CRC. Although previous meta-analyses have reported the carcinogenesis and diagnostic value of F. nucleatum for CRC, studies on the prognosis of F. nucleatum in CRC are few 22,23,24. To the best of our knowledge, this is the first meta-analysis demonstrating the association between F. nucleatum and the clinical and molecular characteristics of CRC, and it is the most comprehensive meta-analysis clarifying the prognostic role of F. nucleatum levels in CRC.

Our results showed a correlation between abundance of F. nucleatum and poor OS in CRC patients. This is similar with a previous study 23, which reports that high level of F. nucleatum in the tumor tissue was associated with poorer OS in CRC patients. However, they found that infection of F. nucleatum was not associated with the DFS and CSS in CRC patients. This was contrary to our study findings. Many evidences have shown that F. nucleatum is associated with CRC development. As a conditional pathogen, F. nucleatum has a high detection rate in metastatic CRC lesions 24.

This is the first meta-analysis demonstrating that high levels of F. nucleatum in CRC tissues were not associated with the tumor side. This contradicts a previous study 25 which reported that the proportion of F. nucleatum-high colorectal cancers gradually increases from the rectum to the cecum. However, another study 14 reported that high F. nucleatum levels had no correlation with the tumor side in CRC patients. Therefore, as F. nucleatum is a critical cancer-promoting factor, our results provided a more convincing evidence to confirm this.

Our results revealed that F. nucleatum had no correlation with the overall TNM stage, but high levels of F. nucleatum were associated with high T and M stages of CRC. Previous experiments had shown that F. nucleatum can activate autophagy pathway-1 by up-regulating CARD3 expression, leading to distant metastasis of tumors 26. Moreover, F. nucleatum can accomplish a series of pathogenic effects by changing the permeability of vascular endothelium 27. This means that F. nucleatum promotes CRC proliferation and distant metastasis via hematogenous metastasis. For more mechanisms, FadA protein, an adhesion molecule of F. nucleatum, can bind to wnt7b E-cadherin on CRC cells and promote F. nucleatum adhesion and invasion of host epithelial cells. Then, F. nucleatum activates β-catenin signaling that regulates expression of related oncogenes and promotes colorectal cancer cell proliferation 28. Moreover, F. nucleatum promotes the expression of several cytokines, such cytokines as IL-6, IL-8, and IL-18, and lead to a proinflammatory microenvironment in CRC which accelerates CRC growth and metastasis 28. Although our meta-analysis results showed a marginal association of high F. nucleatum levels with the higher N stage of CRC, more evidence is needed to clarify the role of F. nucleatum in lymphatic metastasis of CRC. We also found that high levels of F. nucleatum were associated with poorly differentiated of CRC. This was also a result confirmed that F. nucleatum is not only associated with carcinogenesis, but also with poor CRC differentiation.

The accumulation of genetic and epigenetic alterations, influenced from microbial and other environmental exposures and host responses to these exposures, these are all important factors affecting the development of CRC 29. The current study have revealed that high levels of F. nucleatum were related with key tumor molecular features of CRC, including MSI-high and KRAS mutation which have been associated with clinical outcome in advanced CRC. The present data indicate a significant correlation between high F. nucleatum levels and MSI-high from six studies of 2520 patients. MSI status has been proven as a critical predictor for prognosis, and response to chemotherapy or immunotherapy in CRC patients 30-32. A previous study reported the relationship between F. nucleatum and the immune response to CRC by different MSI statuses 33, suggesting that high F. nucleatum levels correlated with MSI status and regulated the antitumor immune response in CRC. Moreover, KRAS mutation also is an important molecular feature for chemotherapy resistance in CRC 34 and high F. nucleatum levels in CRC tissues will cause chemoresistance. Therefore, our results further confirmed this correlation implying that F. nucleatum is another underlying biomarker for the response to immunotherapy and chemotherapy. There was no significant association between high levels of F. nucleatum and mutations of BRAF and PIK2CA, as well as MLH1 hypermethylation in CRC tissues. These results agree with previous studies conducted in single populations, suggesting a role of F. nucleatum mainly with specific mutations in CRC. A more in-depth study of the association between the F. nucleatum levels and other mutations of CRC such as TP53, AKT1, PTEN, and so on, can reveal more biological roles of F. nucleatum in CRC.

Our meta-analysis had some limitations. Firstly, the number of the included studies was relatively small and they were only English studies. This could have resulted in publication bias. Secondly, although sensitivity analysis had been conducted and our results were further confirmed, most of the included studies used qPCR to measure F. nucleatum levels and few used 16S rRNA sequencing. The different methods and cut-off values may cause heterogeneity in some results. Lastly, there were some included studies without reported HRs and 95% CIs in the prognostic outcomes. We attempted to extract survival data from Kaplan-Meier curves according to the previous reported method 10. This may have impacted the precision of the prognostic outcomes of F. nucleatum in CRC.

Despite these shortcomings, there is sufficient evidence to suggest that CRC with high F. nucleatum levels are at high risk of poor prognosis, including OS, DFS, and CSS. Our results also suggest that high F. nucleatum levels are correlated with tumor growth, distant metastasis, poor differentiation, MSI-high, and KRAS mutation in CRC. Future research on the relationship between F. nucleatum and other clinical and molecular characteristics in CRC should be assessed. In addition, understanding more mechanisms of F. nucleatum in the progression of CRC, and whether antibiotic therapy targeting F. nucleatum will help to prolong the prognosis of patients with CRC, will facilitate the identification of more treatment strategies in CRC.

## Supplementary Material

Supplementary materials.

## Figures and Tables

**Table 1:** Characteristics of the included studies

Author	Year	Country	No.of patients		Age(years)		Data collection	Specimens	Test methods	Cut-off value	Stage	Analysis index	Follow-up months	Analysis methods	NOS score
					(mean or median)										
			Fn-high	Fn-low	Fn-high	Fn-low									
Yanglong Chen	2019	China	35	66	58.72±12.638	58.52±10.421	Retro	tissues	qPCR	2^-ΔCt^	II~III	OS	120	MA	5
Kosuke Mima	2016	America	67	917	68.8 ± 8.3	69.2 ± 8.9	Pro	tissues	qPCR	2^-ΔCt^	I~IV	OS, CSS	120	MA, UA	7
Andrew T. Kunzmann	2019	CzechRepublic	61	129	NR	NR	Retro	tissues	qPCR	2^-ΔCt^	I~IV	OS	72	UA	5
Katsuhiko Nosho	2016	Japan	44	463	65.0 ± 12.1	67.3 ± 11.7	Retro	tissues	qPCR	NR	I~IV	NR	NR	NR	4
Hyeon Jeong Oh	2019	South Korea	204	389	>18	>18	Retro	tissues	qPCR	2^-ΔCt^	II~III	DFS	120	MA	6
Yuan Sun	2016	China	118	34	28-84	28-84	Retro	tissues	qPCR	2^-ΔCt^	I~IV	OS	60	UA	5
Zhiliang Wei	2016	China	90	90	NR	NR	Retro	tissues	16sRNA	0.52%	I~IV	OS, DFS	36	MA	5
Yuko Yamaoka	2017	Japan	50	50	62.5 ± 9.8	63.9 ± 14.5	Retro	tissues	droplet digital PCR	NR	I~IV	OS	120	UA	6
Xuebing Yan	2017	China	187	93	NR	NR	Retro	tissues	qPCR	2^-ΔCt^	I~IV	CSS, DFS	60	MA, UA	6
Yongyu Chen	2017	China	61	27	58	59.4	Retro	tissues	16sRNA, FISH	NR	I~IV	NR	NR	NR	4
Bundgaard-Nielsen	2019	Denmark	29	60	71 ±10.1	71 ±10.1	Retro	tissues	16sRNA	NR	I~IV	OS, DFS	96	UA	5
Dae-Won Lee	2018	Korea	64	64	NR	NR	Retro	tissues	qPCR	2^-ΔCt^	II~III	OS, DFS	96	MA	6
Ana Carolina de Carvalho	2019	Brasil	18	134	60.63 ± 13.7	60.63 ± 13.7	Retro	tissues	qPCR	2^-ΔCt^	I~IV	OS, CSS	96	UA	5

*Pro* prospective, *Retro* retrospective. *CRC* colorectal cancer.* qPCR* quantitative polymerase chain reaction, *16sRNA* 16S ribosomal RNA sequencing, *droplet digital PCR* polymerase chain reaction polymerase chain reaction,* qrT-PCR* quantitative reverse transcription polymerase chain reaction, *FISH* fluorescence in situ hybridization.* NR* not report. *OS* overall survival, *DFS disease-free survival*, *MA* multivariate analysis, *UA* univariate analysis.

**Table 2:** Association between high F.nuleatum abundance and Clinical characteristics of patients with CRC

Factors	No. of studies	No. of patients	Pooled OR (95%CI)	*P*-value	Heterogenelty		Statistical method
					I2 (%)	*P*-value	
Tumor side	11	3413	1.26 (0.91, 1.75)	0.17	60%	0.005	Random
TNM Stage	9	3758	1.20 (0.96, 1.51)	0.11	25%	0.22	Fixed
T stage	8	2528	2.20 (1.66, 2.91)	<0.00001	0%	0.65	Fixed
N stage	8	2445	1.27 (0.98, 1.64)	0.07	37%	0.14	Fixed
M stage	3	560	2.11 (1.25, 3.56)	0.005	0%	0.95	Fixed
Differentation	8	2118	1.83 (1.11, 3.03)	0.02	60%	0.01	Random

**Table 3:** Association between high F.nuleatum abundance and molecular characteristic of patients with CRC

Factors	No. of studies	No. of patients	Pooled OR (95%CI)	P-value	Heterogenelty		Statistical method
					I2 (%)	P-value	
MSI	6	2520	2.53 (1.53, 4.20)	0.0003	83%	<0.0001	Random
KRAS	6	2404	1.27 (1.00, 1.61)	0.05	28%	0.23	Fixed
BRAF	6	2499	1.93 (0.91, 4.11)	0.09	65%	0.01	Random
MLH1	3	1211	0.78 (0.06, 9.39)	0.84	94%	<0.0001	Random
PIK2CA	3	1603	1.21 (0.74, 1.97)	0.45	0%	0.86	Fixed

*MSI* microsatellite instability, *KRAS* k-ras gene mutation*, BRAF* b-raf gene mutation,* MLH1* MLH1 hypermethylation, *PIK2CA* PIK3CA gene mutation.

**Table 4:** Sensitivity analysis of studies evaluated F.nuleatum on clinicopathological characteristics of CRC

Factors	No. of studies	No. of patients	Pooled OR (95%CI)	*P*-value	Heterogenelty		Statistical method
					I2 (%)	*P*-value	
Tumor side	8	3035	1.17 (0.77, 1.77)	0.47	67%	0.003	Random
TNM Stage	7	2662	1.05 (0.80~1.36)	0.74	2%	0.41	Fixed
T stage	6	2249	2.14 (1.56, 2.93)	<0.00001	0%	0.46	Fixed
N stage	5	2067	1.01 (0.74, 1.38)	0.94	0%	0.43	Fixed
OS	5	1789	1.36 (1.16, 1.61)	0.0002	0%	0.63	Fixed
DFS	3	1119	1.68 (1.22, 2.32)	0.002	0%	0.74	Fixed

*OS* overall survival, *DFS* disease-free survival.

## Abbreviations

F. nucleatum: Fusobacterium. nucleatum
CRC: colorectal cancer
qPCR: quantitative polymerase chain reaction
16sRNA: 16S ribosomal RNA sequencing
qrT-PCR: quantitative reverse transcription polymerase chain reaction
OS: overall survival
DFS: disease-free survival
CSS: cancer-specific survival
MSI: microsatellite instability
KRAS: k-ras gene mutation
BRAF: b-raf gene mutation
MLH1: MLH1 hypermethylation
PIK2CA: PIK3CA gene mutation
