# Supplementary material for: Clinicopathological and prognostic significance of Fusobacterium nucleatum infection in colorectal cancer: a meta-analysis
Source: J Cancer. 2021 Jan 15;12(6):1583–91. doi: 10.7150/jca.50111 (PMC7890333; doi:10.7150/jca.50111)
Supplement: Supplementary file 1 — Supplementary materials. [file jcav12p1583s1.pdf]

Supplementary Material:

A **Primary tumor side for colorectal cancer: high F.nucleatum versus low F.nucleatum**

Study or Subgroup	Fn-high Events	Fn-high Total	Fn-low Events	Fn-low Total	Weight	Odds Ratio M-H, Random, 95% CI
Ana Carolina de Carvalho 2019	10	18	28	134	6.4%	4.73 [1.71, 13.11]
Andrew T. Kunzmann 2019	22	61	39	129	10.0%	1.30 [0.68, 2.48]
Hyeon Jeong Oh 2019	69	204	115	389	13.6%	1.22 [0.85, 1.75]
Katsuhiko Nosho 2016	19	44	152	467	10.2%	1.57 [0.84, 2.95]
Kosuke Mima 2016	776	835	224	231	8.3%	0.41 [0.19, 0.91]
Xuebing Yan 2017	98	187	52	93	11.9%	0.87 [0.53, 1.43]
Yanglong Chen 2019	5	25	25	66	5.8%	0.41 [0.14, 1.23]
Yongyu Chen 2017	39	61	16	37	8.0%	2.33 [1.01, 5.36]
Yuan Sun 2016	42	118	7	34	7.2%	2.13 [0.86, 5.31]
Yuko Yamaoka 2017	21	50	14	50	8.0%	1.86 [0.81, 4.29]
Zhiliang Wei 2016	35	90	34	90	10.6%	1.05 [0.57, 1.91]
**Total (95% CI)**		**1693**		**1720**	**100.0%**	**1.26 [0.91, 1.75]**
Total events	1136		706			

Heterogeneity: Tau² = 0.17; Chi² = 25.24, df = 10 (P = 0.005); I² = 60%
Test for overall effect: Z = 1.39 (P = 0.17)

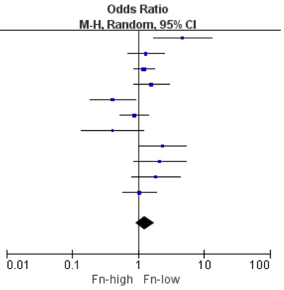

B **TNM Stage for colorectal cancer: high F.nucleatum versus low F.nucleatum**

Study or Subgroup	Experimental Events	Experimental Total	Control Events	Control Total	Weight	Odds Ratio M-H, Fixed, 95% CI
Ana Carolina de Carvalho 2019	15	17	93	134	1.8%	3.31 [0.72, 15.12]
Andrew T. Kunzmann 2019	28	60	66	127	16.5%	0.81 [0.44, 1.50]
Hyeon Jeong Oh 2019	16	204	27	389	12.5%	1.14 [0.60, 2.17]
Katsuhiko Nosho 2016	23	44	272	467	16.4%	0.79 [0.42, 1.46]
Kosuke Mima 2016	27	62	384	915	20.1%	1.07 [0.63, 1.79]
Yanglong Chen 2019	10	25	30	66	7.2%	0.80 [0.31, 2.04]
Yuan Sun 2016	73	118	16	34	6.9%	1.82 [0.85, 3.94]
Yuko Yamaoka 2017	30	50	20	50	5.9%	2.25 [1.01, 5.01]
Zhiliang Wei 2016	48	90	37	90	12.6%	1.64 [0.91, 2.95]
**Total (95% CI)**		**670**		**2272**	**100.0%**	**1.20 [0.96, 1.51]**
Total events	270		945			

Heterogeneity: Chi² = 10.61, df = 8 (P = 0.22); I² = 25%
Test for overall effect: Z = 1.60 (P = 0.11)

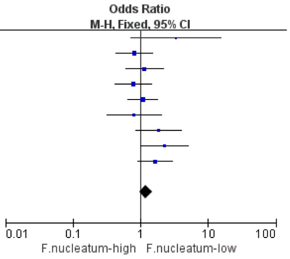

C **T stage for colorectal cancer: high F.nucleatum versus low F.nucleatum**

Study or Subgroup	Fn-high Events	Fn-high Total	Fn-low Events	Fn-low Total	Weight	Odds Ratio M-H, Fixed, 95% CI
Ana Carolina de Carvalho 2019	14	18	85	134	6.3%	2.02 [0.63, 6.47]
Hyeon Jeong Oh 2019	195	204	345	389	14.8%	2.76 [1.32, 5.78]
Kosuke Mima 2016	52	61	625	920	16.2%	2.73 [1.33, 5.61]
Xuebing Yan 2017	132	187	52	92	28.9%	1.85 [1.10, 3.10]
Yanglong Chen 2019	22	25	62	66	5.8%	0.47 [0.10, 2.28]
Yuan Sun 2016	100	118	25	34	8.4%	2.00 [0.80, 4.98]
Yuko Yamaoka 2017	45	50	38	50	5.4%	2.84 [0.92, 8.79]
Zhiliang Wei 2016	75	90	61	90	14.3%	2.38 [1.17, 4.83]
**Total (95% CI)**		**753**		**1775**	**100.0%**	**2.20 [1.66, 2.91]**
Total events	635		1293			

Heterogeneity: Chi² = 5.11, df = 7 (P = 0.65); I² = 0%
Test for overall effect: Z = 5.52 (P < 0.00001)

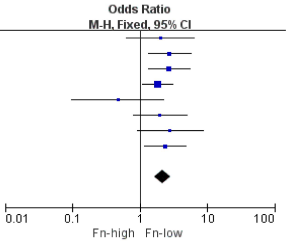

D **N stage for colorectal cancer: high F.nucleatum versus low F.nucleatum**

Study or Subgroup	Fn-high Events	Fn-high Total	Fn-low Events	Fn-low Total	Weight	Odds Ratio M-H, Fixed, 95% CI
Hyeon Jeong Oh 2019	170	204	333	389	36.2%	0.84 [0.53, 1.34]
Kosuke Mima 2016	23	59	326	892	23.4%	1.11 [0.65, 1.90]
Xuebing Yan 2017	187	187	93	93		Not estimable
Yanglong Chen 2019	10	25	30	66	9.4%	0.80 [0.31, 2.04]
Yongyu Chen 2017	7	61	3	37	3.1%	1.47 [0.36, 6.07]
Yuan Sun 2016	35	118	6	34	6.2%	1.97 [0.75, 5.17]
Yuko Yamaoka 2017	27	50	18	50	7.9%	2.09 [0.94, 4.65]
Zhiliang Wei 2016	48	90	31	90	13.7%	2.18 [1.19, 3.97]
**Total (95% CI)**		**794**		**1651**	**100.0%**	**1.27 [0.98, 1.64]**
Total events	507		840			

Heterogeneity: Chi² = 9.60, df = 6 (P = 0.14); I² = 37%
Test for overall effect: Z = 1.84 (P = 0.07)

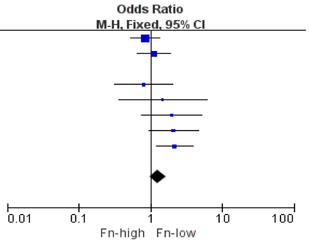

E **Distant metastases for colorectal cancer: high F.nucleatum versus low F.nucleatum**

Study or Subgroup	Fn-high Events	Fn-high Total	Fn-low Events	Fn-low Total	Weight	Odds Ratio M-H, Fixed, 95% CI
Xuebing Yan 2017	49	187	13	93	61.5%	2.19 [1.12, 4.27]
Yuko Yamaoka 2017	13	50	7	50	24.9%	2.16 [0.78, 5.98]
Zhiliang Wei 2016	5	90	3	90	13.6%	1.71 [0.40, 7.36]
**Total (95% CI)**		**327**		**233**	**100.0%**	**2.11 [1.25, 3.56]**
Total events	67		23			

Heterogeneity: Chi² = 0.09, df = 2 (P = 0.95); I² = 0%
Test for overall effect: Z = 2.81 (P = 0.005)

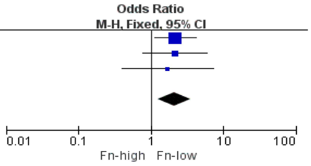

F **Differentiation for colorectal cancer: high F.nucleatum versus low F.nucleatum**

Study or Subgroup	Fn-high Events	Fn-high Total	Fn-low Events	Fn-low Total	Weight	Odds Ratio M-H, Random, 95% CI
Ana Carolina de Carvalho 2019	4	16	7	134	8.5%	6.05 [1.55, 23.65]
Kosuke Mima 2016	19	67	88	1000	17.6%	4.10 [2.31, 7.29]
Xuebing Yan 2017	51	187	23	93	17.6%	1.14 [0.65, 2.02]
Yanglong Chen 2019	4	25	15	66	9.8%	0.65 [0.19, 2.18]
Yongyu Chen 2017	15	61	6	37	11.4%	1.68 [0.59, 4.82]
Yuan Sun 2016	23	117	3	35	9.3%	2.61 [0.73, 9.28]
Yuko Yamaoka 2017	6	50	5	50	9.4%	1.23 [0.35, 4.32]
Zhiliang Wei 2016	27	90	21	90	16.3%	1.41 [0.72, 2.74]
**Total (95% CI)**		**613**		**1505**	**100.0%**	**1.83 [1.11, 3.03]**
Total events	149		168			

Heterogeneity: Tau² = 0.29; Chi² = 17.59, df = 7 (P = 0.01); I² = 60%
Test for overall effect: Z = 2.36 (P = 0.02)

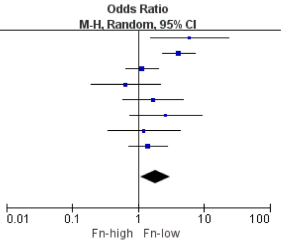

S1. Forest plot of association between F.nuleatum level with clinicopathological characteristics: a. primary tumor site b. TNM Stage c. T stage d. N stage e. distant metastases f. differentiation

A **MSI for coloretal cancer: high F.nucleatum versus low F.nucleatum**

Study or Subgroup	Fn-high Events	Fn-high Total	Fn-low Events	Fn-low Total	Weight	Risk Ratio M-H, Random, 95% CI
Ana Carolina de Carvalho 2019	8	18	13	134	15.3%	4.58 [2.21, 9.51]
Andrew T. Kunzmann 2019	10	50	3	108	9.5%	7.20 [2.07, 25.03]
Hyeon Jeong Oh 2019	15	200	25	385	16.9%	1.16 [0.62, 2.14]
Katsuhiko Nosho 2016	9	44	32	467	16.1%	2.99 [1.52, 5.84]
Kosuke Mima 2016	29	65	136	958	20.8%	3.14 [2.30, 4.30]
Yanglong Chen 2019	21	25	40	66	21.3%	1.39 [1.07, 1.80]
**Total (95% CI)**		**402**		**2118**	**100.0%**	**2.53 [1.53, 4.20]**
Total events	92		249			

Heterogeneity: Tau² = 0.29; Chi² = 29.77, df = 5 (P < 0.0001); I² = 83%
Test for overall effect: Z = 3.61 (P = 0.0003)

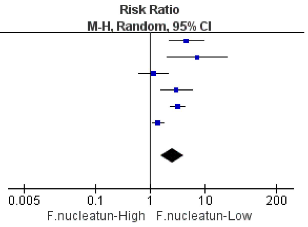

B **KRAS for coloretal cancer: high F.nucleatum versus low F.nucleam**

Study or Subgroup	Fn-high Events	Fn-high Total	Fn-low Events	Fn-low Total	Weight	Odds Ratio M-H, Fixed, 95% CI
Andrew T. Kunzmann 2019	17	35	30	87	7.5%	1.79 [0.81, 3.98]
Hyeon Jeong Oh 2019	67	207	102	388	40.4%	1.34 [0.93, 1.94]
Katsuhiko Nosho 2016	14	44	143	467	14.1%	1.06 [0.54, 2.05]
Kosuke Mima 2016	24	62	399	927	25.8%	0.84 [0.49, 1.42]
Yanglong Chen 2019	14	24	36	66	6.7%	1.17 [0.45, 3.00]
Yuko Yamaoka 2017	25	49	13	48	5.4%	2.80 [1.20, 6.55]
**Total (95% CI)**		**421**		**1983**	**100.0%**	**1.27 [1.00, 1.61]**
Total events	161		723			

Heterogeneity: Chi² = 6.90, df = 5 (P = 0.23); I² = 28%
Test for overall effect: Z = 1.98 (P = 0.05)

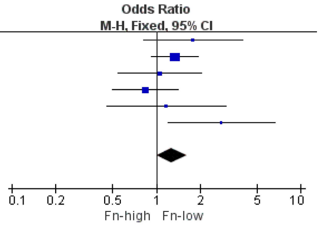

C **BRAF for coloretal cancer: high F.nucleatum versus low F.nucleam**

Study or Subgroup	Fn-high Events	Fn-high Total	Fn-low Events	Fn-low Total	Weight	Odds Ratio M-H, Random, 95% CI
Ana Carolina de Carvalho 2019	5	19	6	132	15.2%	7.50 [2.03, 27.77]
Andrew T. Kunzmann 2019	4	35	6	87	15.0%	1.74 [0.46, 6.59]
Hyeon Jeong Oh 2019	4	204	15	389	17.4%	0.50 [0.16, 1.52]
Katsuhiko Nosho 2016	6	44	22	467	19.3%	3.19 [1.22, 8.35]
Kosuke Mima 2016	21	66	144	965	24.5%	2.66 [1.54, 4.60]
Yanglong Chen 2019	1	25	6	66	8.5%	0.42 [0.05, 3.65]
**Total (95% CI)**		**393**		**2106**	**100.0%**	**1.93 [0.91, 4.11]**
Total events	41		199			

Heterogeneity: Tau² = 0.53; Chi² = 14.13, df = 5 (P = 0.01); I² = 65%
Test for overall effect: Z = 1.71 (P = 0.09)

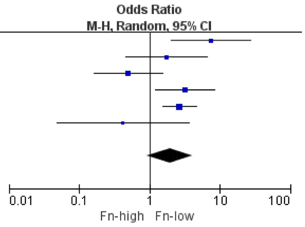

D **MLH1 for coloretal cancer: high F.nucleatum versus low F.nucleam**

Study or Subgroup	Fn-high Events	Fn-high Total	Fn-low Events	Fn-low Total	Weight	Odds Ratio M-H, Random, 95% CI
Ana Carolina de Carvalho 2019	8	15	109	119	32.9%	0.10 [0.03, 0.35]
Kosuke Mima 2016	25	62	109	916	35.0%	5.00 [2.90, 8.63]
Yuko Yamaoka 2017	4	49	5	50	32.1%	0.80 [0.20, 3.17]
**Total (95% CI)**		**126**		**1085**	**100.0%**	**0.78 [0.06, 9.39]**
Total events	37		223			

Heterogeneity: Tau² = 4.53; Chi² = 35.86, df = 2 (P < 0.00001); I² = 94%
Test for overall effect: Z = 0.20 (P = 0.84)

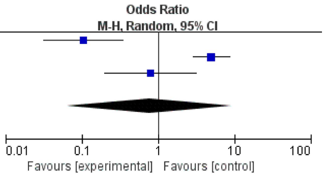

E **MLH1 for coloretal cancer: high F.nucleatum versus low F.nucleam**

Study or Subgroup	Fn-high Events	Fn-high Total	Fn-low Events	Fn-low Total	Weight	Odds Ratio M-H, Fixed, 95% CI
Andrew T. Kunzmann 2019	4	35	9	87	16.4%	1.12 [0.32, 3.90]
Katsuhiko Nosho 2016	7	44	53	467	27.5%	1.48 [0.63, 3.48]
Kosuke Mima 2016	11	63	146	907	56.1%	1.10 [0.56, 2.16]
**Total (95% CI)**		**142**		**1461**	**100.0%**	**1.21 [0.74, 1.97]**
Total events	22		208			

Heterogeneity: Chi² = 0.30, df = 2 (P = 0.86); I² = 0%
Test for overall effect: Z = 0.76 (P = 0.45)

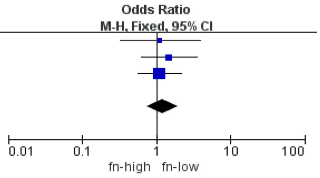

S2. Forest plot of association between F.nuleatum level with molecular characteristics: a. MSI b. KRAS c. BRAF e. MLH1 f. PIK2CA
